# IMPACT OF AGE AND FRAILTY ON THE EFFECT OF A MULTIMODAL INDIVIDUALIZED LONG-TERM INTERVENTION AFTER STROKE: THE LAST-LONG TRIAL

**DOI:** 10.2340/jrm.v58.46250

**Published:** 2026-10-26

**Authors:** Håkon IHLE-HANSEN, Bente THOMMESSEN, Sara Rise LANGLO, Elin BERGH, Anne HOKSTAD, Stian LYDERSEN, Yngve SELJESETH, Ingvild SALTVEDT, Torunn ASKIM

**Affiliations:** 1 Medical Department, Bærum Hospital, Vestre Viken Hospital Trust, Bærum; 2 Department of Neurology, Division of Medicine, Akershus University Hospital, Akershus; 3 Department of Neuromedicine and Movement Science, Faculty of Medicine and Health Science, NTNU-Norwegian University of Science and Technology, Trondheim; 4 Clinic of Rehabilitation, St. Olavs Hospital, Trondheim University Hospital, Trondheim; 5 Regional Centre for Child and Youth Mental Health and Child Welfare, Department of Mental Health, Faculty of Medicine and Health Science, NTNU-Norwegian University of Science and Technology, Trondheim; 6 Department of Medicine, Ålesund Hospital, Helse Møre and Romsdal Hospital Trust, Ålesund; 7 Department of Geriatrics, St. Olavs Hospital, Trondheim University Hospital, Trondheim; 8 Clinic of Internal Medicine, Department of Stroke, St. Olavs Hospital, Trondheim University Hospital, Trondheim Norway

**Keywords:** cerebrovascular disease, long-term follow-up, rehabilitation, community based intervention, multimodal intervention, frailty

## Abstract

**Background:**

The LAST-long randomized trial tested a multidimensional, stroke coordinator-led, community-based intervention and found no overall functional benefit. Because most participants had mild stroke symptoms, whether older or frailer subgroups benefited was examined.

**Methods:**

In this single-blinded randomized controlled trial at 4 Norwegian hospitals (2019–2022), 301 participants were randomized 1:1 to monthly stroke-coordinator meetings for 18 months (structured risk assessment and individualized goals) or to standard care. Primary outcome: modified Rankin Scale (mRS) at 18 months. Secondary outcomes: mortality, cognition, ADLs, physical function, fatigue, and quality of life. *Older age:* ≥ 80 years; frailty: Frailty Index ≥ 0.2. Linear and logistic regression tested age- and frailty-intervention interactions.

**Results:**

mRS data were available for 268 participants (136 intervention, 132 control). Mean age 71.1 (standard deviation 12.4); 66 (24.6%) ≥ 80; 89 (33.2%) frail. Age modified the intervention effect on mRS: among participants ≥ 80, favourable outcome was less likely with intervention vs control (23.4% vs 57.0%, *p* = 0.004). For secondary outcomes, the intervention improved Trail Making Test B in younger participants (−32.2 s, 95% CI −58.0 to −6.4, *p* = 0.015) with a significant age–intervention interaction (*p* = 0.019). No frailty–intervention interaction was found.

**Conclusions:**

Age – but not frailty – modified response to the intervention, indicating differential effects in older participants.

**Clinical Trials.gov identifier:**

NCT03859063

## LAY ABSTRACT

The LAST-long trial evaluated a multidimensional, stroke coordinator-led community intervention and found no overall benefit on functional outcome as measured by the modified Rankin Scale (mRS). Because most participants had experienced mild strokes, we examined whether age and frailty modified the intervention effect. Both older age and frailty were associated with poorer outcomes; however, only age significantly modified the effect on 18-month functional outcome (mRS). Older participants who received the intervention were less likely to achieve an excellent functional outcome, whereas younger participants demonstrated improved executive function, as measured by the Trail Making Test Part B. These findings suggest differential responses to the intervention according to age, and poorer outcomes among older participants, while younger participants appeared to derive cognitive benefits.

Frailty is a clinically identifiable condition of heightened vulnerability caused by age‑related declines across multiple physiological systems, and its prevalence is rising as life expectancy increases in Western populations (1). Importantly, stroke risk and stroke‑related mortality escalate with advancing age, and both older age and frailty are associated with increased mortality and poorer outcomes after acute stroke (1). Advanced age and frailty often coexist, as frailty is strongly age-related, but they may also occur independently. Older age in most stroke studies is defined as ≥ 80 years, whereas there is no universal definition of frailty. Frailty typical manifests as reduced physical function, decreased muscle strength, fatigue, slower gait speed, and unintentional weight loss, all of which increase susceptibility to acute illness (1). Frailty has received limited attention in stroke care, yet studies indicate it is about twice as common in people with a history of stroke compared with those without, with a prevalence of approximately 25–30% among patients with acute stroke (2–4). Frailty, whether present before or after stroke, is associated with lower quality of life, longer hospital stays, greater likelihood of discharge to nursing home care, and reduced chances of functional recovery (5).

Although optimal secondary prevention can reduce the risk of early recurrent vascular events after TIA or stroke by up to 80% (6), adherence to guideline recommendations varies substantially (7). Clinical trials of strategies to improve adherence have shown promising results: motivational interviewing has been associated with improved adherence to pharmacotherapy (8), and tailored counselling may increase post‑stroke participation in physical activity (9). The large heterogeneity in symptoms and rates of decline highlights the need to better define risk profiles and to deliver targeted interventions to limit functional deterioration after stroke. The LAST-long study was the first to test whether monthly meetings with a community-based stroke coordinator, using a multidimensional, patient-centred approach that targeted patients’ unmet needs, could prevent functional decline during long-term follow-up after acute stroke. The intervention was in line with the European Stroke Organization’s *Stroke Action Plan*, which recommends coordinated post‑rehabilitation support and prioritizes research on the transition to life after stroke, including long‑term follow‑up and optimal care models (10). Patients with cognitive, motor, or fatigue-related impairments 3 months after stroke were invited and randomized to either standard follow-up or monthly follow-up with a community-based stroke coordinator for 18 months.

The intervention was not superior to standard care in preventing dependency and functional decline in the overall study population, which had relatively high functional levels at baseline (11). However, subgroups of patients – such as older or frail individuals – might potentially benefit from such a multidimensional and patient-centred approach.

Accordingly, this sub-study aimed to examine whether age or frailty modified the effect of the intervention on functional outcomes and secondary outcomes related to cognition, physical function, and self-reported health.

## METHODS

### Study design and participants

The LAST-long trial was a multicentre, single-blinded randomized controlled trial conducted at 4 hospitals in Norway (2 university and 2 regional), in close collaboration with primary healthcare services in 6 municipalities, from January 1, 2019, to November 30, 2022. The follow-up was completed on August 28, 2024. Participants were randomized in a 1:1 ratio to either a long-term, stroke coordinator-led, patient-centred, multidomain follow-up programme or standard care. All participants in LAST-long received evidence-based stroke unit treatment and rehabilitation according to individual needs after hospital discharge. Standard care follow-up is primarily provided by municipal health services and GPs, with a specialist stroke follow-up typically offered at approximately 3 months. This sub-study of the LAST-long randomized controlled trial is reported in accordance with the Consolidated Standards of Reporting Trials (CONSORT) guidelines (12).

Patients were consecutively recruited at the stroke outpatient clinic after completing the initial rehabilitation phase, approximately 3 months (range 2–4 months) after stroke onset, and were followed for 18 months. The primary outcome was the modified Rankin Scale (mRS) at 18 months.

The study protocol has been published previously (13). In brief, patients were eligible for inclusion if they were aged ≥ 18 years, had an mRS score < 5, a life expectancy exceeding 1 year, no severe comorbidity that would interfere with the study protocol (e.g., severe neurological disease, dementia, or substance abuse, as judged by the study physician), and evidence of functional decline on at least 1 of the tests listed in Table I.

**Table I:** List of screening tests used to identify eligible patients. Eligibility required functional decline on at least 1 of the listed tests

Screening test	Patients were available for the study if they had a score of (indicating functional decline):
Short Physical Performance Battery (SPPB)	Less than 10 points
Montreal Cognitive Assessment (MoCA)	Less than 26 points
Fatigue Severity Scale (FSS-7)	More than 27 points
Hospital Anxiety and Depression Scale (HADS)	More than 7 points on the anxiety items and/or More than 7 points on the depression items
Item 3 on Advanced Hand Activities (Motor Assessment Scale)	Not able to draw 10 horizontal lines within 20 s

### Intervention

The primary objective of the LAST-long trial was to investigate whether an 18-month multimodal personalized intervention delivered by a dedicated stroke coordinator was superior to standard care with regard to dependency. Participants were randomized to either monthly meetings with a newly established community-based stroke coordinator in addition to standard care or standard care only. Rather than targeting organizational or system-level changes, the intervention in our study is individualized and tailored to each patient. Using a study-specific checklist, the coordinator had 2 main roles: (1) to develop an individualized treatment plan and (2) to motivate and assist participants in following this plan by using existing health and social care services. In the present study, the LAST-long checklist was used as a guide for a structured interview to assess the patients’ risk profile within the following domains: (*i*) health and lifestyle, (*ii*) physical function, (*iii*) cognition and mood, and (*iv*) social function (Table SI). The checklist was developed based on the World Stroke Organization (WSO) Post-Stroke Checklist and the Comprehensive Geriatric Assessment (CGA) (14, 15).

For patients identified as being at risk in 1 or more domains, the stroke coordinator and the participant jointly developed an individualized treatment plan based on personal goals, aiming to maintain or improve function. The plan was evaluated and revised at every meeting. Only existing healthcare and community-based services were used to achieve these goals. We did not collect complete, systematic records of the specific treatments delivered to individual participants. An overview of the intervention is provided in Fig. 1, and a more comprehensive description of the study design has been published previously (11, 13).

**Fig. 1:**
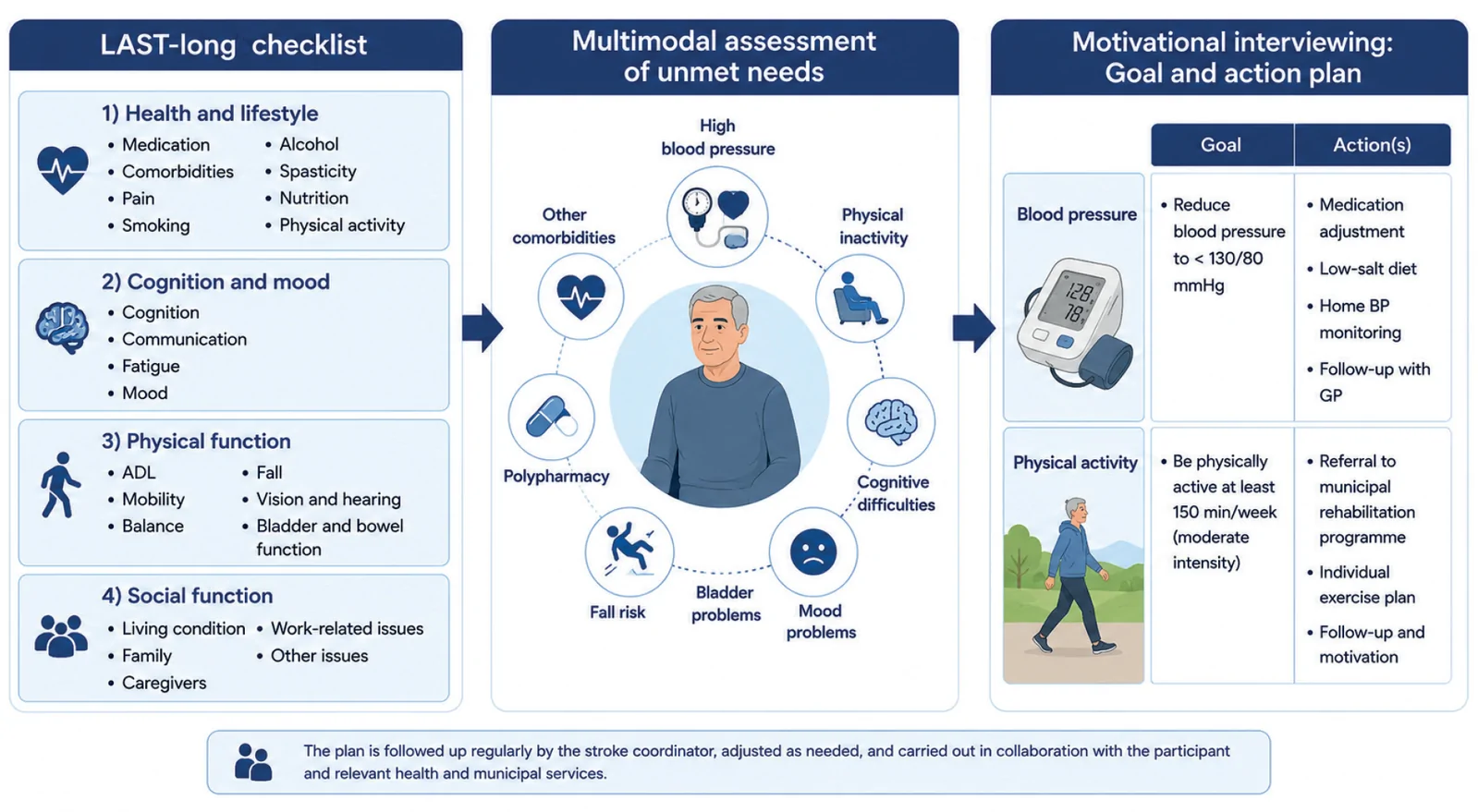
Overview of the study intervention.

In contrast, standard care did not include any structured or fixed follow-up.

### Outcome variables

The primary outcome was the modified Rankin Scale (mRS) score at 18-month follow-up, assessed by a member of the study personnel who was blinded to the treatment allocation. A score of mRS ≤ 1 was considered as an excellent outcome.

For this sub-study, we specifically aimed to examine the effect of the intervention on the following prespecified secondary outcome measures: (*i*) mortality; (*ii*) cognition, assessed with the Montreal Cognitive Assessment (MoCA) and Trail Making Test B (TMT-B) (16, 17); (*iii*) physical performance, assessed with the Short Physical Performance Battery (SPPB) (18); (*iv*) activities of daily living, assessed with the Nottingham Extended Activities of Daily Living (NEADL) scale (19); and (*v*) patient-reported outcomes focusing on fatigue and quality of life, assessed with the Fatigue Severity Scale (FSS-7) and EQ-5D-5L Index (20, 21) (Table II).

**Table II:** Overview of outcome variables

Test	Description	Range and cut-offs
mRS	Six-point scale of person’s functional independence in daily activities after stroke	A score of 0–1 is considered as an excellent outcome, while 6 is equal to death
Mortality	Death occurring between study entry and 18-month follow-up	Defines as mRS equal to 6
MoCA	Global cognitive screening test – comprising several cognitive domains including executive functioning, which is prone to vascular pathology, designed to detect mild cognitive impairment	Score ranging from 0–30, and higher score denotes better cognition; score of 26 or higher is considered normal
SPPB	Screening test of physical function among older people, comprising 3 sub-tests (balance, walking speed, and chair raising test)	Score ranging from 0–12, a score of 10 or above is considered normal, while a score belove 8 indicates emerging decline in ADL functions
NEADL	Questionnaire that includes 22 items concerning everyday activities, all scored on a 4-point scale (0–3)	Score ranging from 0–66; higher score indicates greater independence
FFS-7	Seven-item questionnaire validated for measuring fatigue across various conditions	Each question is score from 1–7; 1 denotes no fatigue; mean score per item of 4 or greater indicates fatigue; maximum total score 49
EQ-5D-5L Index	Five-dimension (mobility, self-care, usual activities, pain/discomfort and anxiety/depression) questionnaire on self-experienced health; each dimension again has 5 levels: no problems (1p) to extreme problems (5p)	Index score ranges from 0 to 1, with 1 indicating perfect health

### Other variables

Demographic and clinical variables, pre-stroke function, comorbidities, medication use, and relevant information from the index stroke hospital stay were collected at the time of study inclusion. This included the National Institutes of Health Stroke Scale (NIHSS) score assessed on hospital admission. Data were obtained through structured interviews, questionnaires, blood samples, and review of hospital records. The exploratory variables were age and frailty. Older age was defined as ≥ 80 years (< 80 vs ≥ 80). Frailty was assessed using the Frailty Index (FI), which is based on the accumulation of a broad range of variables, including the domains comorbidity, activity in daily living, cognition, nutrition, and physical function (22, 23). Each item was scored as 0 (no deficit) or 1 (deficit present), with the exception of MoCA, grip strength, and walking speed, which were assigned point scores of 0, 0.3, 0.7, or 1 depending on performance. The total number of points is summed and divided by the number of assessed items to obtain a ratio representing the degree of frailty. A higher score indicates a greater level of frailty. In this study, an FI ratio of ≥ 0.2 was classified as frailty. The list of variables accounted for in the calculation of the Frailty index is presented in Table III.

**Table III:** List of domains with subitems included in the Frailty Index

Comorbidity	Reduced level of functioning (activity of daily living): “needs help with”	Physical function	Cognition	Nutrition
Hypertension	Transportation	Walking speed*	MoCA^§^	BMI < 21 kg/m^2^
Atrial fibrillation	Housekeeping	Grip strength^¶^		
Coronary heart disease	Use of smartphone	Walking distance		
Heart failure	Writing/reading			
Diabetes	Grocery			
Hearing or visual impairment	Economics			
Fall(s) within the last year	Climbing stairs			
Cancer last 5 years	Cooking			
Obstructive pulmonary disease				
Chronic kidney disease				

Frailty categories based on the Frailty Index (CGA-FI): For each item, a score of either 0 (no problems) or 1 (problem present) is assigned. By summing the total number of points and dividing by the number of assessed items, a ratio is obtained that describes the degree of frailty: Non-frail (< 0.10), Pre-frail (0.10–0.19); Mild frailty (0.20–0.29); Moderate frailty (0.30–0.39); Severe frailty (≥ 0.40).

* Walking speed: ≥ 1.0 m/s = 0 points; 0.80–0.99 m/s = 0.3 points; 0.60–0.79 m/s = 0.7 points; < 0.60 m/s = 1 point.

¶ Grip strength: 0 points: ≥ 32.0 kg (men)/≥ 20.0 kg (women); 0.5 points: 26.0–31.9 kg (men)/16.0–19.9 kg (women); 1 point: < 26.0 kg (men)/< 16.0 kg (women).

§ MoCA score: 24–30 = 0 points; 22–23 = 0.3 points; 19–21 = 0.7 points; < 19 = 1 point.

### Data management and statistical analysis

Based on the intention-to-treat population from the LAST-long study, our study population included all patients with a valid mRS score at the 18-month follow-up. Descriptive statistics are presented as mean and standard deviation (SD), or number and percentage, as appropriate.

We used logistic regression analyses with the primary outcome (mRS) or the secondary outcome (mortality), both at 18 months, as dependent variables. The main covariates were intervention and age and their interaction, or intervention and frailty and their interaction. Similarly, the effect of age and frailty on the remaining secondary outcomes at 18 months (MoCA, SPPB, Nottingham I-ADL, EQ-5D-5L Index, and FSS-7) was evaluated using linear regression with the same covariates. All regression analyses were adjusted for the predefined covariates sex, National Institutes of Health Stroke Scale (NIHSS) score on hospital admission, study site, and pre-stroke mRS.

A sensitivity analysis using ordinal logistic regression was performed with the 18-month mRS as an ordinal outcome.

Results are presented as regression coefficients for linear regression, odds ratios (ORs) for logistic regression, and relative risk ratios (RRRs) for ordinal logistic regression, with corresponding 95% confidence intervals (CIs). The significance level was set at *p* < 0.05 for all analyses. In the secondary outcome analyses, *p*-values from the adjusted models (14 tests for interaction in total) were corrected for multiple testing using the Benjamini–Hochberg procedure to control the false discovery rate (FDR), using the software R (R Foundation for Statistical Computing, Vienna, Austria).

All other statistical analyses were performed using Stata version 18 (StataCorp, College Station, TX, USA).

## RESULTS

### Study characteristics

Of the 301 patients who were included (152 in the intervention and 149 in the control group) in the LAST-long trial, 268 (89.0%) (136 in intervention and 132 in the control group) had a valid mRS at 18 months and were included in this study. The mean (SD) age at inclusion was 71.1 (12.4 [range 33 to 92]) years, and 66 (24.6%) (35 in the intervention and 31 in control group) were above the age of 80, of whom 35 (53.0) were considered frail. Frailty was present in 89 (33.2%) (44 in the intervention and 45 in the control group) of the participants.

Older participants and those classified as frail had a higher burden of cardiovascular risk factors and a lower pre-stroke functional level, reflected by higher mean (SD) premorbid mRS scores (1.0 [0.9] vs 0.6 [0.5] for older vs younger participants; and 1.1 [1.0] vs 0.5 [0.8], for frail vs non-frail participants). Neither older age nor frailty was associated with stroke aetiology or stroke subtype. Frailty, but not older age, was associated with greater stroke severity, as measured by NIHSS at hospital admission. Patient characteristics according to age and frailty are presented in Table IV. Descriptive statistics for the outcome variables at study inclusion and after 18 months according to age and frailty are presented in Table V.

**Table IV:** Patient characteristics by age and frailty

Factor	Age ≥ 80 (*n* = 66)	Age < 80 (*n* = 202)	Frail (*n* = 89)	Non-frail (*n* = 179)
Age (years), mean (SD)	84.7 (3.2)	66.7 (11.0)	77.4 (9.6)	68.1 (12.5)
≥ 80	N/A	N/A	35 (39.3)	31 (17.3)
Frail, *n* (%)	35 (53.0)	54 (26.7)	N/A	N/A
Education				
Higher education, *n* (%)	27 (40.9)	93 (46.0)	30 (33.7)	90 (50.3)
Female sex, *n* (%)	41 (62.1)	82 (40.6)	48 (53.9)	75 (41.9)
Stroke characteristics				
NIHSS admission, *n* (%)				
< 8	59 (89.4)	172 (85.2)	64 (71.9)	167 (93.3)
8–16	7 (10.6)	20 (9.9)	19 (21.4)	8 (4.5)
> 16	0 (0.0)	10 (5.0)	6 (6.7)	4 (2.2)
Stroke type, *n* (%)				
Infarction	61 (92.4)	184 (91.1)	80 (89.9)	165 (92.2)
Haemorrhage	5 (7.6)	15 (7.4)	9 (10.1)	11 (6.2)
Unknown (neg MR/CT)	0 (0.0)	3 (1.5)	0 (0.0)	3 (1.7)
TOAST classification, *n* (%)				
Large-artery atherosclerosis	13 (19.7)	35 (17.3)	19 (21.4)	29 (16.2)
Cardio embolism	15 (22.7)	33 (16.3)	17 (19.1)	31 (17.3)
Small-vessel occlusion	15 (22.7)	40 (19.8)	15 (16.9)	40 (22.4)
Stroke of other determined aetiology	1 (1.5)	16 (31.2)	3 (3.4)	14 (7.8)
Stroke of undetermined aetiology	17 (25.8)	63 (31.2)	26 (29.2)	54 (30.2)
Haemorrhage	5 (7.6)	15 (7.4)	9 (10.1)	11 (6.2)
Pre-stroke mRS, mean (SD)	1.0 (0.9)	0.6 (0.8)	1.1 (1.0)	0.5 (0.8)
Pre-stroke mRS, *n* (%)				
modified Rankin Scale 0–1	49 (74.2)	165 (81.7)	61 (68.5)	153 (85.5)
modified Rankin Scale ≥ 2	17 (25.8)	37 (18.3)	28 (31.5)	26 (14.5)
Pre-stroke comorbidity, *n* (%)				
Cerebral infarction	14 (21.2)	33 (16.3)	24 (27.0)	23 (12.9)
Cerebral haemorrhage	5 (7.6)	7 (3.5)	5 (5.6)	7 (3.9)
Transient ischaemic attack	9 (13.6)	16 (7.9)	11 (12.4)	14 (7.8)
Myocardial infarction	10 (15.2)	24 (11.9)	18 (20.2)	16 (8.9)
Heart failure	4 (6.1)	4 (2.0)	6 (6.7)	2 (1.1)
Atrial fibrillation	18 (27.3)	22 (10.9)	22 (24.7)	18 (10.1)
Hypertension	42 (63.6)	98 (48.5)	60 (67.4)	80 (44.7)
Hypercholesterolemia	18 (27.3)	72 (35.6)	32 (35.4)	58 (32.4)
Diabetes	9 (13.6)	35 (17.3)	21 (23.6)	23 (12.9)
Kidney disease	8 (12.1)	7 (3.5)	9 (10.1)	6 (3.4)
At inclusion (3 months post stroke)				
Time from stroke (days), mean (SD)	113.2 (17.8)	106.6 (18.1)	111.8 (16.8)	106.4 (18.7)
Living situation, *n* (%)				
Alone	38 (57.6)	52 (25.7)	35 (39.3)	55 (30.7)
Frailty index, *n* (%)				
Non-frail (< 0.10)	11 (14.5)	107 (47.6)	N/A	118 (59.3)
Pre-frail (0.10–0.19)	24 (31.6)	57 (25.3)	N/A	81 (40.7)
Mild frailty (0.20–0.29)	27 (35.5)	44 (19.6)	71 (69.6)	N/A
Moderate frailty (0.30–0.39)	9 (11.8)	10 (4.4)	19 (18.6)	N/A
Severe frailty (≥ 0.40)	5 (6.6)	7 (3.1)	12 (11.8)	N/A
mRS, mean (SD)	1.9 (0.9)	1.6 (0.9)	2.3 (0.9)	1.3 (0.7)
mRS, *n* (%)				
mRS 0–1	25 (37.9)	103 (51.0)	18 (20.2)	110 (61.5)
mRS ≥ 2	41 (62.1)	99 (49.0)	71 (79.8)	69 (38.5)
Body mass index, mean (SD)*	25.4 (3.8)	27.3 (5.9)	26.3 (5.3)	27.1 (5.7)
Randomized to intervention, *n* (%)	35 (53.0)	101 (50.0)	44 (49.4)	92 (51.4)

NIHSS: National Institutes of Health Stroke Scale; SD: standard deviation; MRI: magnetic resonance imaging; CT: computed tomography.

* Missing 23.

**Table V:** Outcomes variables at baseline and 18 months according to age and frailty

Factor	Age ≥ 80	Age < 80	Frail	Non-frail
Baseline, mean (SD)	*n* = 66	*n* = 202	*n* = 89	*n* = 179
MoCA score*	22.2 (4.0)	25.0 (3.6)	22.6 (4.7)	25.11 (3.1)
Trail making Test B ^¥^ (sec)	188.3 (89.7)	128.1 (76.0)	182.5 (97.8)	124.7 (69.6)
EQ-5D-5L index	0.8 (0.2)	0.8 (0.2)	0.8 (0.2)	0.9 (0.1)
Fatigue Severity Scale (FSS-7)*	2.9 (2.0)	3.7 (2.0)	3.7 (2.0)	3.4 (2.1)
Nottingham Extended ADL*	51.0 (12.8)	54.3 (11.9)	41.5 (13.0)	59.4 (5.5)
Short Physical Performance Battery^¶^	9.0 (2.9)	10.2 (2.5)	7.8 (3.1)	10.9 (1.6)
18 month follow-up (alive)	*n* = 61	*n* = 199	*n* = 82	*n* = 178
Excellent mRS (0–1), *n* (%)	24 (36.4)	110 (54.5)	25 (28.1)	109 (60.9)
Mortality, *n* (%)	5 (7.6)	3 (1.5)	7 (7.9)	1 (0.6)
MoCA score^χ^	23.5 (4.6)	26.1 (3.5)	24.3 (4.2)	26.1 (3.6)
Trail making Test B^β^ (sec)	186.0 (94.7)	127.7 (79.8)	181.4 (104.5)	121.0 (70.3)
EQ-5D-5L index^§^	0.8 (0.2)	0.9 (0.1)	0.8 (0.1)	0.9 (0.1)
Fatigue Severity Scale (FSS-7)^Ψ^	2.9 (1.7)	3.7 (1.9)	3.9 (1.8)	3.4 (1.9)
Nottingham Extended ADL^µ^	51.8 (13.3)	57.4 (11.5)	46.0 (12.1)	60.8 (6.8)
Short Physical Performance Battery^#^	8.6 (2.6)	9.8 (2.5)	7.5 (2.7)	10.4 (2.00)

Values are *n* (%) unless stated otherwise. MoCA Montreal Cognitive Assessment.

Missing baseline *1, ^¥^33, ^¶^3.

Missing 18 months (minus the 8 who died): ^§^28, ^Χ^59, ^β^78, ^Ψ^36, ^µ^28, ^#^59. Missing values were evenly distributed between the control and intervention groups; however, the proportion of missing values was higher among older and frailer participants.

*Effect of age on intervention and outcomes.* Older age significantly modified the effect of the intervention on the main outcome (mRS) (OR 0.17, 95% CI 0.05–0.63; interaction *p* = 0.008). Specifically, older participants (≥ 80 years) in the intervention group had a substantially lower probability of an excellent outcome (mRS 0–1) compared with the control group (23.4% vs 57.0%, *p* = 0.004), whereas no effect was observed among younger patients (*p* = 0.69).

For the secondary outcome, age modified the effect of intervention on cognitive performance as measured by the TMT B (interaction *p* = 0.019). Among younger participants, the intervention was associated with significantly shorter completion time (–32.2 s, 95% CI –58.0 to –6.4, *p* = 0.015). In contrast, older participants in the intervention group used non-significant longer time compared with their counterparts in the control group (43.9 s, –14.1 to 101.8, *p* = 0.137). For mortality, MoCA, SPPB, FSS-7, EQ-5D-5L Index, and Nottingham ADL scores, no age–intervention interactions reached statistical significance (Table VI and Table SII).

**Table VI:** Model-adjusted estimated probabilities and means

Factor	Age ≥ 80 years	Age < 80 years	Frail	Non-Frail
mRS 0–1 estimated probabilities (%) *n/N*^§^				
Intervention	23.4* 8/24	53.8 57/110	36.2 14/25	51.0 51/109
Control	57.0* 16/24	53.1 53/110	31.4 11/25	64.5 58/109
Mortality estimated probabilities (%) *n/N*^Ψ^				
Intervention	2.7 1/5	2.3 1/3	6.0 3/7	Not estimable 0/1
Control	10.7 4/5	1.0 2/3	8.3 4/7	1.4 1/1
MoCA (estimated mean score)				
Intervention	23.3	26.4	24.3	26.4
Control	24.0	25.6	25.0	25.5
TMT B (estimated mean sec.)				
Intervention	206.9	112.6^¥^	163.2	115.4
Control	163.0	144.8^¥^	189.6	131.1
NEADL (estimated mean score)				
Intervention	53.1	57.9	54.6	57.8
Control	54.0	56.2	54.9	56.2
SPPB (estimated mean score)				
Intervention	8.2	9.8	7.5	10.3
Control	8.8	9.8	8.4	10.1
FSS-7 (estimated mean score)				
Intervention	2.6	4.0	3.7	3.7
Control	2.9	3.5	3.9	3.2
EQ-5D-5L Index (estimated mean index)				
Intervention	0.70	0.70	0.63	0.73
Control	0.72	0.71	0.64	0.74

Adjusted for sex, stroke severity (according to National Institutes of Health Stroke Scale), study site, and pre-morbid mRS. mRS: modified Rankin scale, MoCA: Montreal Cognitive Assessment, TMT B: Trail Making Test B, SPPB: Short Physical Performance Battery, NEADL: Nottingham Extended Activities of Daily Living Scale, FFS-7: 7-item version of the Fatigue Severity Scale.

§ Number of patients with an excellent outcome by age, frailty, and treatment group.

Ψ Number of patients who died by age, frailty, and treatment group.

* *p* for interaction = 0.008,

¥ *p* for interaction = 0.019.

Benjamini–Hochberg-corrected value (FDR): ^¥^*p* for interaction=0.247.

In sensitivity analyses using an ordinal regression model, the results were consistent with the main analysis, demonstrating a significant interaction between treatment and age (RRR = 0.17, *p* = 0.008). Older participants in the intervention group had a lower probability of an excellent outcome (mRS 0–1) (OR 0.18, 95% CI 0.05 to 0.57, *p* = 0.004). Pre-stroke mRS and admission NIHSS remained strong independent predictors of worse mRS scores.

*Effect of frailty on intervention and outcomes.* Frailty was associated with lower odds of an excellent functional outcome but did not modify the effect of the intervention on the main outcome (mRS) (interaction *p* = 0.145).

Across secondary outcomes, frailty consistently predicted poorer outcomes – including slower TMT B performance, lower SPPB score, and lower EQ-5D-5L Index values. However, no significant frailty-intervention interaction were observed in any of the models (see Table VI and Table SII).

The sensitivity analysis using ordinal logistic regression showed that frailty independently predicted worse functional outcomes (*p* < 0.001), while the frailty–intervention interaction remained non-significant (*p* = 0.145), confirming that results were robust to model specification.

## DISCUSSION

In this sub-study of the LAST-long trial, age – but not frailty – modified the effect of the intervention on functional outcome (mRS) at 18 months. Older participants in the intervention group had a significantly lower probability of achieving an excellent outcome. Across secondary outcomes, age also modified the effect of the intervention on Trail Making Test Part B performance, although this association was no longer significant after adjustment for multiple testing. In the frail subgroup, no differences were observed between the intervention and control groups for either the primary or secondary outcomes.

Older participants seemed to derive less benefit from the intervention. This finding may reflect random variation, or it may indicate that older individuals are less able to cope with a comprehensive, long-term intervention. The intensity of the intervention may have been unfavourable, or potentially even detrimental, for this group, or older participants may not have responded adequately to the intervention. This aligns with the hypothesis that complex long-term follow-up interventions may impose stress or fatigue on very old patients (24, 25). In contrast, similar effect modification was not observed for frailty, despite small differences in performance between older and frail participants.

Consistent with a subgroup analysis from the main trial, we found that the intervention improved performance on TMT-B. TMT-B is a measure of executive function, a cognitive domain closely linked to cerebrovascular disease. Improvements in executive function have previously been reported primarily in interventions that include physical exercise and predominantly among younger individuals, which aligns with our findings (26). The absence of a corresponding effect on the MoCA may reflect its broader weighting of cognitive domains beyond executive function. Moreover, although the LAST-long checklist included an assessment of unmet cognitive needs, prior research suggests that cognitive deficits after stroke are frequently under-recognized and under-treated, indicating a potential gap that our intervention may not have fully addressed (27). This may partly explain the lack of improvement in MoCA scores. Age-related reductions in neuroplasticity may partly explain the lack of positive effects among older participants in our cohort.

Comprehensive geriatric assessment is used not only to identify frailty but also to detect potentially modifiable domains that may be optimized to improve outcomes. Frailty is generally considered to be only partially modifiable (1). Despite this, we were unable to demonstrate that stroke-coordinator-led patient-centred, long-term follow-up conferred benefits for frail patients compared with controls. Several explanations are possible, including limited statistical power, insufficient intervention intensity, suboptimal targeting of frailty-related deficits, or reduced reversibility of frailty in this population.

These findings indicate that the overall neutral result of the LAST-long trial cannot be attributed solely to the recruitment of relatively young and healthy participants. Importantly, frail and older participants appeared to derive no benefit from the intervention compared with non-frail or younger individuals. Our findings suggest that future interventions targeting older individuals should be specifically adapted to their functional capacity and care needs to better address individual needs and avoid potential unintended effects. Overall, our results align with prior work which indicated that structured post‑stroke follow‑up can improve some functional outcomes (28), but the evidence is inconsistent and sparse regarding differences by age or frailty (29). On balance, our study does not support routine implementation of a community‑based stroke coordinator, as we observed no overall benefit and no evidence of benefit among frail or older participants.

Our results confirm existing evidence that age and frailty is associated with poorer outcomes. A recent meta-analysis found that frailty was associated with 1.2 higher odds of poor functional outcome after stroke (30), and in the Nor-Coast study the Frailty Index was identified as a strong predictor (OR 4.1) of mRS 3–6 (31).

Few previous studies have assessed the association between frailty and quality of life after stroke; however, our results showing that the frail participants had worse self-perceived health compared with the non-frail are consistent with earlier studies (32).

### Strengths and limitations

The major strengths of the study are the randomized-controlled design, a well-characterized population, including a multidimensional assessment allowing estimation of level of frailty, recruitment from both regional and university hospitals across 4 regions of Norway, as well as a long-term intervention lasting 18 months with good adherence to the study protocol.

Several limitations should be acknowledged. First, this was a sub-study of the LAST-long trial, and the findings should therefore be considered hypothesis-generating. Multiple testing was performed, and after adjustment, the age–intervention interaction for TMT-B was no longer statistically significant. The Frailty Index was calculated at study entry, 3 months after stroke, making it difficult to distinguish between underlying biological frailty and residual post-stroke disability. Furthermore, missing values for secondary outcomes were, as expected, more frequent among older and frailer participants; however, the absolute number was low and should be taken into account when interpreting the results. Excellent rather than good functional outcome according to the mRS was selected because the study population consisted largely of patients with mild strokes and good premorbid functional status, which particular may have limited the potential effect of the intervention on some of the secondary outcome variables. However, whether excellent functional outcome is an equally relevant endpoint among older and frailer individuals is debatable, and the chosen outcome measures may have disadvantaged these groups. Moreover, the absence of information on adherence to individualized goals and treatment plans limits the interpretation of the results.

In addition, the study population consisted predominantly of participants with mild stroke severity. This participant profile may have created a potential ceiling effect, limiting the scope for measurable improvement. Consequently, the findings may not be generalizable to patients with more severe strokes. Finally, the study was conducted within a well-developed healthcare system with universal access, which may have attenuated the observed intervention effects and limits generalizability.

### Conclusion

In this sub-study of the randomized controlled LAST-long trial, age – but not frailty – modified the effect of the intervention on functional outcome at 18 months, with older participants in the intervention group having lower odds of a favourable outcome. Further studies focusing on the needs of older and frail individuals in stroke rehabilitation are warranted.

## Supplementary Material



## Data Availability

In line with the ethical approval, restrictions apply to the availability of data until January 2033. However, after that date, anonymous individual data can be available upon request to the corresponding author.

## Patient involvement

We collaborated with 2 user organizations – Landsforeningen for slagrammede and LHL Hjerneslag og Afasi – in the LAST-long project and held 2 regular meetings each year.
