# Supplementary material for: IMPACT OF AGE AND FRAILTY ON THE EFFECT OF A MULTIMODAL INDIVIDUALIZED LONG-TERM INTERVENTION AFTER STROKE: THE LAST-LONG TRIAL
Source: J Rehabil Med. 2026 Oct 26;58:46250. doi: 10.2340/jrm.v58.46250 (PMC13717427; doi:10.2340/jrm.v58.46250)
Supplement: Supplementary file 1 [file JRM-58-46250-s1.pdf]

Table SI: The domains and subdomains of the LAST-long checklist

1.Health and lifestyle	2. Physical function	3. Cognition and mood	4. Social function
Physical activity	Activities of daily living	Cognition	Living conditions
Medication	Mobility, balance and risk of falling	Communication	Family
Smoking, alcohol and drugs	Vision and hearing	Fatigue	Caregivers
Nutrition	Bladder and bowel function	Mood	Work related matters
Pain			Any other matters
Spasticity			
Comorbidities			

Table SII: P values-for interaction

Outcome	Age-intervention interaction	Frailty-intervention interaction
mRS	0.008	0.145
Mortality	0.167	Not estimable
MoCA	0.268	0.153
TMT B	0.019	0.694
NEADL	0.413	0.550
SPPB	0.478	0.128
FSS-7	0.132	0.227
EQ-5D-5L Index	0.847	0.977

mRS modified Rankin scale, MoCA Montreal Cognitive Assessment, TMT B Trail Making Test B, SPPB Short Physical Performance Battery, Nottingham Extended Activities of Daily Living Scale, FFS-7 7-item version of the Fatigue Severity Scale
